# PLW: Probabilistic Local Walks for detecting protein complexes from protein interaction networks

**DOI:** 10.1186/1471-2164-14-S5-S15

**Published:** 2013-10-16

**Authors:** Daniel Lin-Kit Wong, Xiao-Li Li, Min Wu, Jie Zheng, See-Kiong Ng

**Affiliations:** 1 Data Analytics Department, Institute for Infocomm Research, Agency for Science, Technology and Research (A*STAR), Singapore 138632, Singapore; 2 School of Computer Engineering, Nanyang Technological University, Singapore 639798, Singapore; 3 Genome Institute of Singapore, Agency for Science, Technology and Research (A*STAR), Singapore 138672, Singapore

## Abstract

**Background:**

Many biological processes are carried out by proteins interacting with each other in the form of protein complexes. However, large-scale detection of protein complexes has remained constrained by experimental limitations. As such, computational detection of protein complexes by applying clustering algorithms on the abundantly available protein-protein interaction (PPI) networks is an important alternative. However, many current algorithms have overlooked the importance of selecting seeds for expansion into clusters without excluding important proteins and including many noisy ones, while ensuring a high degree of functional homogeneity amongst the proteins detected for the complexes.

**Results:**

We designed a novel method called Probabilistic Local Walks (PLW) which clusters regions in a PPI network with high functional similarity to find protein complex cores with high precision and efficiency in O (*|V| *log *|V| *+ *|E|*) time. A seed selection strategy, which prioritises seeds with dense neighbourhoods, was devised. We defined a topological measure, called common neighbour similarity, to estimate the functional similarity of two proteins given the number of their common neighbours.

**Conclusions:**

Our proposed PLW algorithm achieved the highest F-measure (recall and precision) when compared to 11 state-of-the-art methods on yeast protein interaction data, with an improvement of 16.7% over the next highest score. Our experiments also demonstrated that our seed selection strategy is able to increase algorithm precision when applied to three previous protein complex mining techniques.

**Availability:**

The software, datasets and predicted complexes are available at http://wonglkd.github.io/PLW

## Background

Protein complexes are physical aggregations of proteins that interact with each other at the same location and time. They are a cornerstone of many critical cellular processes, providing the molecular machinery to perform a vast spectrum of complex biological functions. Some important examples include the nuclear pore complexes for regulating the passage of proteins and RNA between the nucleus and cytoplasm [1] and the proteasomes for breaking down unneeded or damaged proteins [2]. Elucidating these important protein complexes is critical for understanding cellular function and structure. In fact, many proteins are functional only when assembled into a protein complex [3-5].

Unfortunately, biologists have yet to overcome the many experimental limitations for the large-scale detection of protein complexes, such as the shortcomings of Tandem Affinity Purification (a common wet lab complex detection method) listed in a recent protein complex survey paper [6]. As a result, only a tiny fraction of the possible protein complexes have been confirmed by wet lab experiments.

In contrast, high-throughput methods for detecting pairwise protein interactions (e.g., yeast two-hybrid screening) have enabled the interactomes of many organisms to be mapped efficiently, yielding large scale protein-protein interaction datasets that are readily available in public databases for data mining and knowledge discovery. Given the experimental limitations of large scale detection of protein complexes, computational methods for detecting protein complexes from the rich protein-protein interaction datasets present a useful alternative.

By modelling a protein-protein interaction (PPI) network as an undirected graph, where a vertex denotes a unique protein and an edge represents an interaction between two proteins, we can expect protein complexes to manifest graphically in the PPI networks as cliques. In practice, given that data derived from high-throughput screening techniques are often incomplete (i.e. have missing interactions) and noisy (i.e. have wrong interactions that do not actually occur in the cell) [7], the protein complexes are more likely to manifest in the PPI networks as dense regions with many interactions (dense subgraphs) than as cliques (fully connected subgraphs -- all proteins in a complex interact with each other) [8]. Many protein complex prediction algorithms are cognisant of this and search for regions with high density. This is often done by expanding seeds into maximally dense subgraphs where a seed is a small group of vertices (commonly a single vertex or a triangle) [9].

The MCODE algorithm proposed by Bader *et al. *[10] was one of the first methods to mine PPI networks for protein complexes in this fashion. It scored vertices by their neighbourhood densities, selected those seeds with high scores, and then traversed the graph outwards from each seed to recursively include other highly scored vertices to form clusters. However, MCODE is known for predicting too little complexes with too many proteins in each predicted complex [6]. Simulating random walks in graphs is a fast and robust method for clustering network data [7], and has been applied to detect protein complexes in PPI networks. The Markov Cluster Algorithm (MCL) [11,12] popularised this technique but had limitations such as being unable to detect overlapping protein complexes and predicting noisy clusters [13]. Algorithms such as SR-MCL [14], MCL-CA [13,15] and RRW [16] were proposed to overcome these limitations; however, SR-MCL still predicted too many complexes while the RRW model was too rigid and predicted complexes of a particular size (69% of the complexes predicted by RRW contained five proteins).

We can exploit the graph theoretic properties of the biological structures of protein complexes for better complex detection in PPI networks. A protein complex generally contains a core in which proteins are highly co-expressed and share high functional similarity. The protein complex is often surrounded by attachments, which are proteins that assist the core to perform subordinate functions [17]. The core-attachment architecture of experimentally detected protein complexes was demonstrated by Gavin *et al. *[5]. A few algorithms, e.g., COACH [17], CORE [18], MCL-CA [13] and CACHET [19], have employed this model to predict biologically meaningful complexes. These algorithms typically consist of two major steps: 1. detect protein complex cores, and 2. add other proteins that are closely associated with the core as attachments. The demonstration of modularity in yeast PPI networks [5] has also led to the application of modularity optimisation in protein complex detection by finding regions that are relatively denser compared to their surroundings [20]. While this approach is able to detect the less dense protein complexes, existing modularity functions have limitations such as the modularity resolution limit [21] and misidentification [22].

In all these approaches, finding high quality seeds to expand without excluding important proteins or including too many noisy ones in the seeds is pivotal to increasing the algorithms' precision. In addition, given that proteins within a protein complex interact with each other to perform a common biological function, the algorithms should also focus on ensuring that the protein members detected as protein complexes have high functional homogeneity. In this paper, we propose a Probabilistic Local Walks (PLW) algorithm to detect protein complexes. We devise a seed selection strategy and formulate a topological measure called common neighbour similarity to estimate the functional similarity in two proteins. Using these, we illustrate how PLW performs probabilistic local walks efficiently to mine protein complex cores by identifying areas of high common neighbour similarity. The effectiveness of common neighbour similarity is established through its high correspondence to functional similarity. Finally, we validate PLW using yeast PPI data and show that it significantly outperforms 11 existing methods for complex prediction in terms of various evaluation metrics (e.g., F-measure).

## Methods

In this section, we present a novel Probabilistic Local Walks (PLW) algorithm to mine a PPI network/graph *G_ppi _*for protein complexes. This PPI graph is formally defined as the undirected graph *G_ppi _*= (*V_ppi_*, *E_ppi_*) where *E_ppi _*= {(*u*, *v*)*|u*, *v *∈ *V_ppi_*}. Our proposed PLW algorithm consists of three main steps:

1. selecting proteins that are located in a dense region and have high degree centrality as seeds,

2. expanding these seeds to find protein complex cores through iterative probabilistic local walks, and

3. adding attachment proteins that are closely linked to the cores.

Since a complex core is the "heart" of a protein complex, it should be a subgraph that satisfies the two following structural graph-theoretic properties.

First, given that protein members of a complex core highly interact with each other, it should be *dense*. Let us define a subgraph *G*' = (*V*', *E*'), where *V*' ⊆ *V_ppi _*and *E*' = {(*u*, *v*)*|*(*u*, *v*) ∈ *E_ppi_*, *u*, *v *∈ *V*'}. We quantify the density of this subgraph using the local clustering coefficient, which is the number of edges *|E*'*| *divided by the theoretical maximum number of edges possible for the graph, *|V*'| * (*|V*'*| - *1)*/*2.

**Definition 1**. The density of the graph G' = (V', E') is defined as:

(1) $$density\left(G^{\prime }\right)=\frac{2*|E^{\prime }|}{\left|V^{\prime }|*|V^{\prime }-1\right|}$$

Secondly, it has been observed that there is a high degree of functional homogeneity in experimentally-verified protein complex cores where proteins work together and share common biological functions [5,17]. As such, we also require that the member proteins of a protein complex core should have many *common neighbours *or interact with a similar set of proteins. We postulate that protein A and B are likely to possess similar functions if protein A shares a number of interaction partners (C, D, ...) with protein B-since A and B can bind to the same proteins, they are likely to share common biochemical and physical properties.

We will define a topological protein similarity measure called *common neighbour similarity *in Equation (5) to quantify the degree of similarity between two proteins by considering the number of common neighbours.

### Seed selection

Choosing high quality protein seeds for expansion is also critical. Most protein complex prediction algorithms have employed a form of local search to expand seeds by including proteins located in the seeds' local neighbourhood graph. However, if a complex does not exist in the neighbourhood of these seeds, the algorithm will never be able to find the complex regardless of the quality of the local search method. Furthermore, low quality seeds may also result in a false positive complex being detected. For example, if a protein on the periphery of multiple complexes is chosen as a seed, the resulting predicted complex may subsume the multiple complexes under an unrealistic *big false complex *that can not match with any real protein complex.

Let us first provide a number of definitions for seed selection. Given a vertex, its neighbour set and degree are defined as follows.

**Definition 2**. For each vertex v ∈ V_ppi_, the set of its neighbours (or adjacent vertices) is denoted as N_v _= {u|u ⊆ V_ppi_, (u, v) ⊆ E_ppi_}. v's degree in V_ppi _is denoted by deg(v) = |N_v_|.

Given a vertex *v_i _*∈ *V_ppi_*, its local neighbourhood graph $G_{v_{i}}$ is the subgraph formed by *v *and its adjacent vertices (direct neighbours) and the interactions between these proteins, as defined below.

**Definition 3**. For each vertex v_i _∈ V_ppi_, its local neighbourhood graph $G_{v_{i}}=\left(V_{v_{i}},E_{v_{i}}\right)$, where $V_{v_{i}}=\left\{v_{i}\right\}\cup \left\{v|v\in V_{ppi},\left(v,v_{i}\right)\in E_{ppi}\right\},\;E_{v_{i}}=\left\{\left(v_{j},v_{k}\right)|\left(v_{j},v_{k}\right)\in E_{ppi},v_{j},v_{k}\in V_{v_{i}}\right\}$.

We devise the following score function that would identify protein seeds likely to be inside protein complexes, and which have high centrality in those complexes.

**Definition 4**. The score of a seed v_i _is defined as the product of the seed's degree and its neighbourhood graph density.

(2) $$score\left(v_{i}\right)=deg\left(v_{i}\right)*density\left(G_{v_{i}}\right)$$

**Figure 1:**
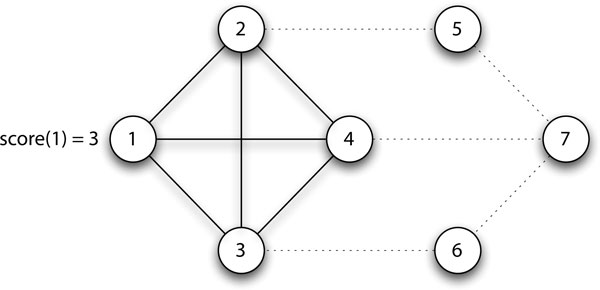
Seed Score (Degree * Neighbourhood Density). The solid edges depict vertex 1's neighbourhood. As *deg*(1) = 3 and $density\left(G_{1}\right)=\frac{6}{0.5*4*3}=1$, *score*(1) = 3 * 1 = 3.

The seed score function takes both degree centrality and neighbourhood graph density into consideration for prioritising the proteins for seeds. We demonstrate its calculation for an example network in Figure 1.

Let us discuss two specific scenarios to illustrate the usefulness of the score function:

1. Given two proteins with the same neighbourhood graph density but different degrees, the protein with the higher degree is more likely to be in a protein complex core as it interacts with more proteins and therefore more likely to serve as key players or coordinators within complex cores, whereas the protein with the lower degree is more likely to be an attachment or on the periphery of a core.

2. Given two proteins with the same degree but different neighbourhood graph density, the protein with the lower neighbourhood graph density might be interacting with proteins from multiple complexes since the connectivity between its neighbours is lower, e.g., vertex 7 in Figure 1. In contrast, a high neighbourhood graph density reflects a high degree of functional homogeneity within the seed's neighbourhood which indicates a higher likelihood of the seed being in a protein complex core, e.g., vertex 1 in Figure 1.

Proteins with higher seed scores are therefore more likely to be in complex cores and should be subsequently expanded to form cores and corresponding complexes. In this paper, we rank proteins by their seed scores and select a fraction, denoted as λ, to be expanded into cores. For example, if λ = 0.3, the top-ranked 30% of proteins are selected as the seed set *V_seeds_*. This selection is formally defined in Equation (3) using *x*, the number of proteins selected; the seed set is defined in Equation (4).

(3) $$x=\left\lfloor \lambda *\left|V\;\right|\right\rfloor ,\;\lambda \in \left(0,\text{1}\right]$$

(4) $$V_{seeds}=\left\{v_{i}|v_{i}\in V_{ppi},\;score\left(v_{i}\right)\text{ are top }x\text{ out of all the proteins in }V_{ppi}\right\}$$

### Core mining using iterative Probabilistic Local Walks (PLW)

Protein complexes have a high degree of functional similarity between their member proteins. Unfortunately, it is infeasible to directly use functional information (say from Gene Ontology) for protein complex core detection, as experimentally verified functional information may not be available for many proteins.

#### Common neighbour similarity

We define a vertex *common neighbour similarity *measure to estimate the functional similarity of two proteins using a topological characteristic, the number of common neighbours. A high number of common neighbours means that the two proteins interact with a similar group of proteins. As the biological function of proteins is determined by the nature of their interactions with other proteins and which proteins they interact with, the number of common neighbours is a good proxy in the absence of functional data. If two protein share a number of interaction partners, they are likely to share biological functions as they could have common biochemical or physical properties to allow them to bind to their common neighbours. In fact, proteins with high vertex *common neighbour similarity *might even be substitutes for each other since they are able to interact with the same set of proteins to carry out similar or identical biological functions.

**Definition 5**. Vertex common neighbour similarity is defined as the cosine similarity of the vector representations of the proteins' neighbourhoods.

(5) $$common\text{\_}neighbour\text{\_}similarity\left(v,u\right)=\left|V_{v}\cap V_{u}\right|/\sqrt{\left|V_{v}|*|V_{u}\right|}$$

Each protein *v_i _*is represented as a vector $V_{v_{i}}$ with a dimension equal to $\left|V_{ppi}\right|$ where an element in $V_{v_{i}}$ is equal to 1 if the corresponding vertex interacts with *v_i _*and 0 otherwise.

**Figure 2:**
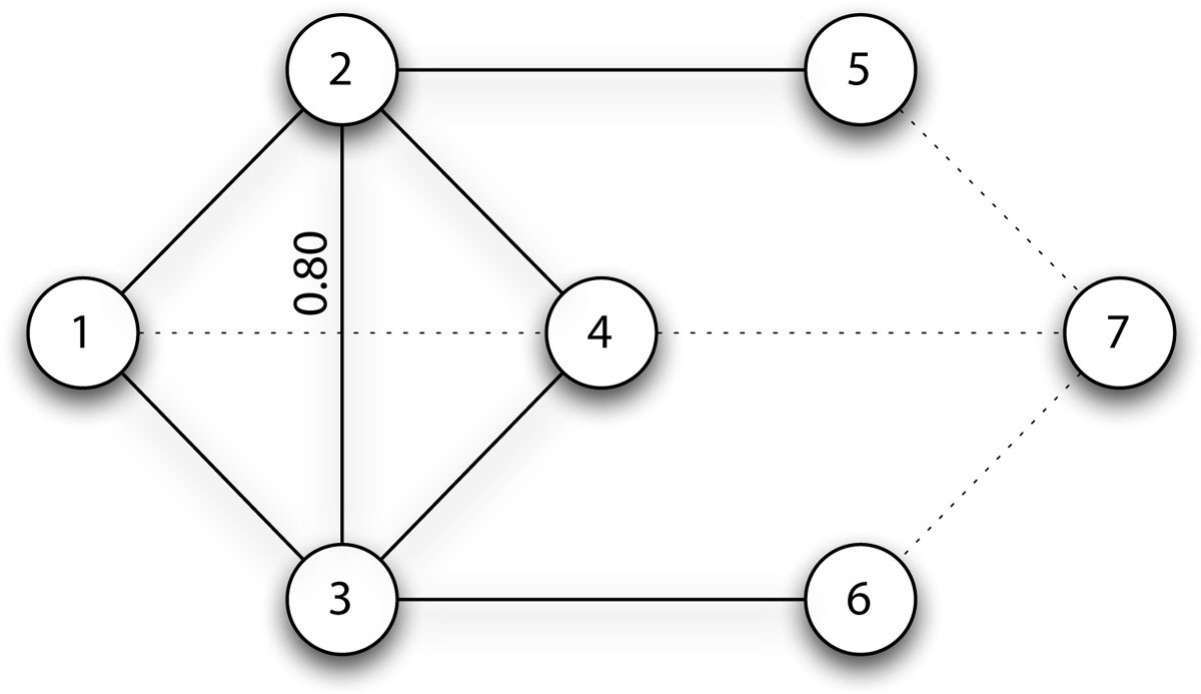
**Common Neighbour Similarity**. The solid edges show the common neighbours of vertices 2 and 3. Vertices 2 and 3 share 2 common neighbours (vertices 1 and 4) and identical neighbourhood sizes, and thus have a high common neighbour similarity of $\frac{2+2}{\sqrt{\left(4+1\right)\left(4+1\right)}}=0.8$. The numerator of (2 + 2) means 2 common neighbours plus the two proteins themselves.

Vertex *common neighbour similarity *can also be calculated using the number of common neighbours normalised by the geometric mean of the neighbourhood size of vertex *u *and *v *as shown in Figure 2. Proteins are more similar if they have a high number of common neighbours and have a similar neighbourhood size. The intuitiveness of this measure in representing functional similarity can be seen in its independent derivation by Goldberg *et al. *and Mete *et al. *[23,24].

#### Basis for Probabilistic Local Walks (PLW)

We propose a novel Probabilistic Local Walks (PLW) algorithm, which will identify for each seed *s *∈ *V_seeds _*proteins that are similar in terms of *common neighbour similarity*, in the vicinity of the seed and which may not be directly connected to the seed by an edge.

**Figure 3:**
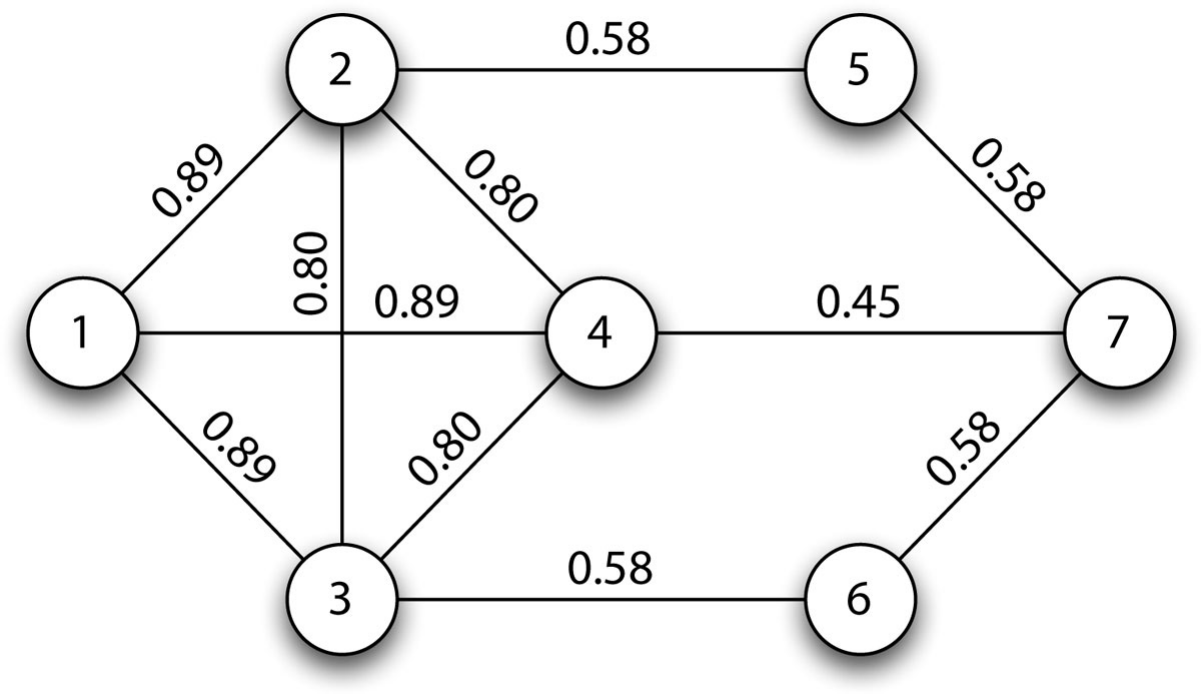
**Example network to illustrate probabilistic local walks**. Edge labels show the common neighbour similarity of the two vertices. The random walker's next step is determined by a weighted random choice, e.g., at vertex 1, the probability of travelling to vertex 2 is $\frac{0.89}{0.89+0.89+0.89}=\frac{1}{3}$.

**Favouring similar proteins using a weighted random choice**. The PLW algorithm takes into account the network structure by favouring edges connecting proteins with higher *common neighbour similarity *for inclusion in the same complex core. This weighted random choice is achieved by choosing the next protein in the walk with probability proportional to the common neighbour similarity between the current protein and each candidate neighbour. Given a protein *v *and its neighbour *u*, we define the probability of walking from *v *to *u *in Equation (6) and provide an illustrated example in Figure 3.

(6) $$P\left(u\right)=\frac{common\text{\_}neighbour\text{\_}similarity\left(v,u\right)}{\sum_{\left(v,p\right)\in E_{ppi}}common\text{\_}neighbour\text{\_}similarity\left(v,p\right)}$$

According to Equation (6), the random walker will pick edges that connect proteins with high common neighbour similarity with a higher probability, and will tend to walk within groups of proteins with high similarity. Performing these probabilistic walks allows us to detect regions of high functional similarity. Making a probabilistic choice instead of greedily choosing the most similar neighbour lessens the chance of getting stuck in local maxima. While a probabilistic local walk can be seen as a finite Markov chain, they are different from the random walks simulated in existing algorithms [11,13,14,16].

In order to perform our proposed PLW algorithm, we transform our *G_ppi _*into a weighted graph *G_sim_*:

**Definition 6**. G_sim _is defined as the graph where each edge (u, v) ∈ E_ppi _has the weight 1 - common_neighbour_similarity(u, v).

(7) $$Gsim=\;\left(V_{sim},\;E_{sim}\right),\;where\;V_{sim}=V_{ppi}$$

(8) $$E_{sim}=\left\{\left(u,v\right)|\left(u,v\right)\in E_{ppi},weight\left(u,v\right)=\text{1}-common\text{\_}neighbour\text{\_}similarity\left(u,v\right)\right\}$$

**Identifying proteins in the vicinity of a seed**. In our PLW algorithm, we ensure that proteins chosen are close to the seed in the PPI network by limiting the length of the walk using a starting energy *α *and penalty *γ*. Each probabilistic walk starts with an energy of *α*. For each step taken, *γ *= 1 *- common_neighbour_similarity*(*v, u*) is deducted from the walk's energy, where *v *is the current vertex and *u *is the next vertex to be visited. The walk terminates when taking the next step would cause the energy to fall below 0. The penalty term penalises walking to dissimilar proteins by reducing the length of the walk. This limits the reachable vertices to the *α*-vicinity of the seed, which is defined as follows:

**Definition 7**. The α-vicinity of a seed s is defined as the set of vertices for which the distance to s on Gsim is less than or equal to α. The distance is the length of the shortest path between the two vertices.

(9) $$vicinity\left(s,\alpha \right)=\left\{u|u\in V_{ppi},distance_{G_{sim}}\left(s,u\right)\leq \alpha \right\}$$

*α *was chosen by estimating the diameter (length of longest shortest path) of protein complex cores. We set *α *to 2.00 to cover direct neighbours as well as neighbours of neighbours, as there may be missing interactions (false negatives) between a seed and fellow proteins in the same complex core. Indeed, 88.2% of complexes in the CYC2008 manually-curated yeast complex catalogue [25] have a diameter of at most 2 in the DIP PPI dataset (connected complexes with at least three proteins were considered in this calculation).

Compared to existing work RRW [16], which uses conventional random walks with restarts that potentially allow the walk to traverse the entire graph, our proposed PLW algorithm does not allow for proteins that are distant in the PPI graph to be detected in the same complex core. This better models the detection of protein complex cores, since proteins are highly unlikely to be in the same core as distant proteins. We thus avoid generating the giant protein complexes that are predicted by existing techniques such as MCODE [10].

#### Implementation of the Probabilistic Local Walks (PLW) algorithm

Our PLW algorithm can be implemented in two parts:

1. performing probabilistic local walks and counting how frequently each vertex is visited in walks starting from a seed *s *(demonstrated in Algorithm 1), and

2. identifying the core vertices for each seed by evaluating the statistical significance of their visit frequency counts (demonstrated in Algorithm 2).

**Collate visit frequency counts**. Algorithm 1 illustrates the calculation of *visitCount*(*s*, *v_j_*), which is the frequency count that a vertex *v_j _*is visited from the seed *s*. For each seed *s*, we expand the seed *w *times for *w *probabilistic local walks, with *w *set to 100 for this paper. Lines 3-14 represent one walk (one iteration).

For each probabilistic local walk starting at a seed *s*, we initialise the current vertex to be the seed *s *with an initial energy of *α *in lines 3 and 4. In lines 5-14, the algorithm walks from vertex to vertex until the energy falls below 0. At each non-seed vertex that it visits, it increments *visitCount*(*s*, *v*). It then picks the next vertex to visit using the weighted random choice described in the previous section. The algorithm applies the penalty term γ (in lines 10-12) to limit its graph traversal to the seed's *α*-vicinity. We bound γ to be a minimum of 0.01 in line 11 to ensure termination of the walk even when similarity is high (*>*0.99).

Table 1 shows one probabilistic local walk (lines 3-14 of Algorithm 1) on the graph in Figure 3. If the random walker travels from vertex 1 to vertex 2, its energy will deplete by γ = 1 *- *0.89 = 0.11. Should the random walker choose to traverse the vertices 1, 2, 3, 4, 2, 3, 4, 2, 3, 4, 7 in that order, its energy will progress from *α *(2.00 in this paper) to a final value of *-*0.26. Note that *visitCount *is cumulative over the *w *walks.

**Table 1:** Possible outcome of a probabilistic local walk on the network in Figure 3.

Steps Taken	*v *(Current Vertex)	Energy Left	*γ *(Energy Penalty)
0	1	2.00	-
1	2	1.89	1 *- *0.89 = 0.11
2	3	1.69	1 *- *0.80 = 0.20
3	4	1.49	1 *- *0.80 = 0.20
4	2	1.29	1 *- *0.80 = 0.20
5	3	1.09	1 *- *0.80 = 0.20
6	4	0.89	1 *- *0.80 = 0.20
7	2	0.69	1 *- *0.80 = 0.20
8	3	0.49	1 *- *0.80 = 0.20
9	4	0.29	1 *- *0.80 = 0.20
10	7	*-*0.26	1 *-*0.45 = 0.55

At the end of this walk, *visitCount*(1, 2) = 3, *visitCount*(1, 3) = 3 and *visitCount*(1, 4) = 3 (assuming this is the first walk taken from this seed). Note that vertex 7 is not visited as it would cause the energy to become negative.

**Algorithm 1 **Compute *visitCount *using probabilistic walks

1: **function **ComputeVisitCount(*s*)

2:  **for ***i ← *1, *w ***do **▶ Perform *w *walks

3:   *v ← s *▶ Initialise random walk at *s*

4:   *energy ← α *▶ Initialise *energy *at *α *(2.00 in this paper)

5:   **repeat**

6:     **if ***v*≠*s ***then**

7:       *visitCount*(*s, v*) *← visitCount*(*s, v*) + 1 ▶ Record visit to vertex *v*

8:     **end if**

9:     select *u *randomly from *N_G_*(*v*) with *P*(*u*) *∝ common_neighbour_similarity*(*v*, *u*)

      ▶ Make a weighted random choice in line 9

10:   γ *← *1 *- common_neighbour_similarity*(*v, u*) ▶ Compute penalty for traversing edge (*v, u*)

11:   γ *← *max(γ, 0.01) ▶ Ensure termination when *similarity*(*v, u*) = 1

12:   energy ← energy - γ

13:       v ← u

14:     **until ***energy <*0

15:   **end for**

16: **end function**

**Identification of protein complex cores**. Algorithm 2 demonstrates how the protein complex cores are formed using *visitCount*. We calculate the standard scores for all ln(*visitCount*(*s_i_*, *v_j _*))∀*visitCount*(*s_i_, v_j_*) ≠ 0, and select statistically significant *ln*(*visitCount*(*s_i_*, *v_j_*) values in line 3 using a significance level of 0.5%. We apply a logarithmic transformation in lines 2, 3 and 6 to lessen the impact of outliers. This is a common method of improving the normality of variables [26].

For each seed *s *∈ *V_seeds_*, we find the significant vertices for walks starting from *s *and select them to form the complex core (in line 6). We discard duplicate cores as well as cores with two or less proteins, since detecting two-protein cores is more dependent on the interaction data quality than the clustering method [6].

**Algorithm 2 **Identify cores using recorded *visitCount*

1:   **function **MineCores(*V_seeds_*)

2:   Calculate Z-scores of all ln(*visitCount*(*s, v*))*∀visitCount*(*s, v*) ≠ 0

3:   Calculate statistical significance of all ln(*visitCount*(*s*, *v*)) ▶ *p *= 0.5% is used for this paper

4:   *cores *← ∅

5:   **for all ***s *∈ *V_seeds _***do**

6:     *candidateCore *← {*s*} ∪ {*v|v ∈ V_ppi_*, ln(*visitCount*(*s, v*)) is significant}

7:     **if **|*candidateCore*| > 2 **then**

8:       *cores *← *cores *∪ *candidateCore*

9:     **end if**

10:   **end for**

11: **end function**

### Adding of attachments

We select proteins that interact with more than half of the proteins in the core as attachments. The neighbourhood of a complex core *C *= (*V_C_*, *E_C_*) is defined as *N*(*C*) = {*u|*(*u*, *v*) ∈ *E_ppi_*, *v *∈ *V_C_*, *u *∈ *V_ppi_*, *u ∈ VC*}. *N*(*C*) consists of the direct neighbours of the vertices in *C *connected with *v*. *|N_v _∩ V_C_| *is the number of proteins in the core that are also neighbours of *v*. By selecting only attachments with $\frac{\left|N_{v}\cap V_{C}\right|}{\left|V_{C}\right|}>0.5$, we ensure that they are closely associated and interact closely with proteins in the protein complex core.

### Overall PLW algorithm

The overall PLW algorithm, which combines all the major steps, is shown as follows in Figure 3. This includes seed selection in lines 2-3, core mining in lines 4-7 and adding of attachments in lines 9-15.

The time complexity of our PLW algorithm is $O\left(n\text{ log }n+m\right)$, where *n *= *|V_ppi_| *and *m *= *|E_ppi_|*. This allows PLW to compete on large-scale PPI networks that can not be handled by the majority of existing methods [27]. Sorting the seeds for seed selection takes $O\left(n\text{ log }n\right)$ time. The weighted random choices can be precomputed for all vertices in $O\left(n+m\right)$ time. Expanding the seeds into cores takes x * w * q operations, where *x *is the number of seeds selected for expansion into cores, *w *is the number of probabilistic local walks taken and *q *is the average number of steps taken. Given that *w *and *q *are constants (100 and 2.22 respectively in our paper) and *x *is at most *n*, the expansion of the cores takes $O\left(n\right)$ time.

**Algorithm 3 **Overall PLW Algorithm for Mining Protein Complexes

1: **function **MineComplexes(*G_ppi _*= (*V_ppi_*, *E_ppi_*))

2:   $x\leftarrow \left\lfloor \lambda *|V_{ppi}|\right\rfloor$ ▶ Seed selection in lines 2-3

3:   *Vseeds ← *vertices in *Vppi *with the *x *highest scores

4:   **for all ***s *∈ *V_seeds _***do **▶ Core mining in lines 4-7

5:     ComputeVisitCount(*s*) ▶ See Algorithm 1 for details

6:   **end for**

7:   *cores ← *MineCores(*Vseeds*) ▶ See Algorithm 2 for details

8:   *clusters *← ∅

9:   **for all ***sg *∈ *cores ***do **▶ Add attachments in lines 9-15

10:     **for all ***v *∈ *Vppi\sg ***do**

11:       E_sg,v _← {(v, u)|(v, u) ∈ E_ppi_, u ∈ sg}

          ▶ *E_sg,v _*are the edges connecting *v *and the core *sg*

12:     **end for**

13:     *sg *← *sg *∪ {*v|v *∈ *V_ppi_*, |*E_sg,v _*|/|*sg*| > 0.5}

14:     *clusters *← *clusters *∪ *sg*

15:   **end for**

16:     **return ***clusters*

17: **end function**

## Results and discussion

We performed extensive experiments to illustrate the effectiveness of our proposed PLW algorithm. We first present our experimental datasets and evaluation metrics, followed by our results.

### Experimental datasets

We applied our proposed PLW algorithm on two experimental yeast PPI datasets. One was retrieved from the Database of Interacting Proteins (DIP) [28] and was used in [17]. Another is a combined dataset of experimentally-determined PPIs that was used in [29]. This dataset combines PPIs from six experiments, namely [30], [4], [5], [31], [32] and [33], and is hereafter referred as "COMBINED6" for convenience. To evaluate the seed selection strategy, we used an additional yeast PPI dataset from the BioGRID database [34], which was used in [35]. It was not used for the main comparative evaluation as a significant number of algorithms could not run in time on this larger dataset.

After we removed duplicated edges and self-loops, the DIP dataset contains 17,201 interactions among 4,930 yeast proteins, the COMBINED6 dataset contains 17,327 interactions among 3,861 yeast proteins and the BioGRID dataset contains 59,748 interactions among 5,640 yeast proteins,

Two sets of protein complexes were utilised as gold standards to validate the predicted protein complexes. The first set is the CYC2008 catalogue of manually curated protein complexes from Wodak's lab [25]. The second set used in [36,37] (denoted as "NewMIPS") was derived from three sources: MIPS [38], Aloy *et al. *[39] and the Gene Ontology (GO) annotations in the SGD database [40]. Complexes smaller than 3 proteins were filtered out from both benchmarks. After this step, there are 236 complexes left in the CYC2008 and 328 complexes in NewMIPS. For the CYC2008 benchmark, the largest complex is the cytoplasmic ribosomal large subunit with 81 proteins and the average size of the complexes is 6.68 proteins.

### Evaluation metrics

Let *P *and *B *be the set of predicted complexes and the set of benchmark complexes. We apply the neighbourhood affinity score to quantify the degree of overlap between a predicted cluster *p *∈ *P *and a benchmark complex *b *∈ *B*, denoted as *N A*(*p*, *b*) in Equation (10). A predicted cluster *p *is considered to match a complex *b *if *N A*(*p*, *b*) *≥ *ω. ω is set as 0.2 in our experiments and the same setting was used in [6,9,10,17,41].

(10) $$NA\left(p,b\right)=\frac{|p\cap b{|}^{2}}{\left|p|*|b\right|}$$

*N_cp _*in Equation (11) is defined as the number of predicted complexes that match at least one benchmark complex and *N_cb _*in Equation (12) to be the number of benchmark complexes that match at least one predicted complex.

(11) $$N_{cp}=\left|\{,p\right|p\in P,\exists b\in B,NA\left(p,b\right)\geq \omega \}|$$

(12) $$N_{cb}=\left|\{,b\right|b\in B,\exists p\in P,NA\left(p,b\right)\geq \omega \}|$$

Based on the above definitions of *N_cp _*and *N_cb_*, we use Recall, Precision and F-measure (the harmonic mean of Recall and Precision) in Equation (13) and Equation (14) to evaluate overall algorithm performance.

(13) $$Precision=\frac{N_{cp}}{\left|P\right|},Recall=\frac{N_{cb}}{\left|B\right|}$$

(14) $$F-Measure=\frac{2*Precision*Recall}{Precision+Recall}$$

In addition, sensitivity (*Sn*), positive predictive value (*PPV*) and geometric accuracy (*Accuracy*) have recently been proposed to evaluate the quality of protein complex predictions [7,36,42]. Given *n *benchmark complexes (*B*) and *m *predicted clusters (*P*), let *T_ij _*denote the number of common proteins between the *i^th ^*benchmark complex (*b_i_*) and *j^th ^*predicted cluster (*p_j _*), i.e. *T_ij _*= *|b_i _∩ p_j_|*. *Sn*, *PPV *and *Accuracy *are then defined in Equation (15). Generally, a high *Sn *indicates that the predicted complexes have a good coverage of the proteins in the benchmark complexes. High *PPV *values indicate that the predicted complexes are likely to be true positives.

(15) $$Sn=\frac{\sum_{i}max_{j}T_{i,j}}{\sum_{i}|b_{i}|},PPV=\frac{\sum_{j}max_{j}T_{i,j}}{\sum_{j}|\cup \left(b_{i}\cap p_{j}\right)|},Accuracy=\sqrt{Sn*PPV}$$

### Performance comparison with existing methods

We compared the performance of PLW with 11 state-of-the-art methods on DIP data. These methods are: MCODE [10], RNSC [43], MCL [11,12], DPClus [44], CFinder [45], CMC [29], RRW [16], COACH [17], SPICi [27], SR-MCL [14] and ClusterONE [35].

We set the parameters of each algorithm to the authors' recommended values. For instance, the inflation parameter in MCL was set as 1.9 on DIP data [37] and the minimum cluster size of RRW was set to 5 [16]. Please note that we removed predicted clusters of two or less proteins. For a fair comparison, we did not supply biological data to algorithms that supported them (e.g., GO annotations) as most of these techniques focused on the topological properties of PPI networks.

#### F-measure and geometric accuracy

**Figure 4:**
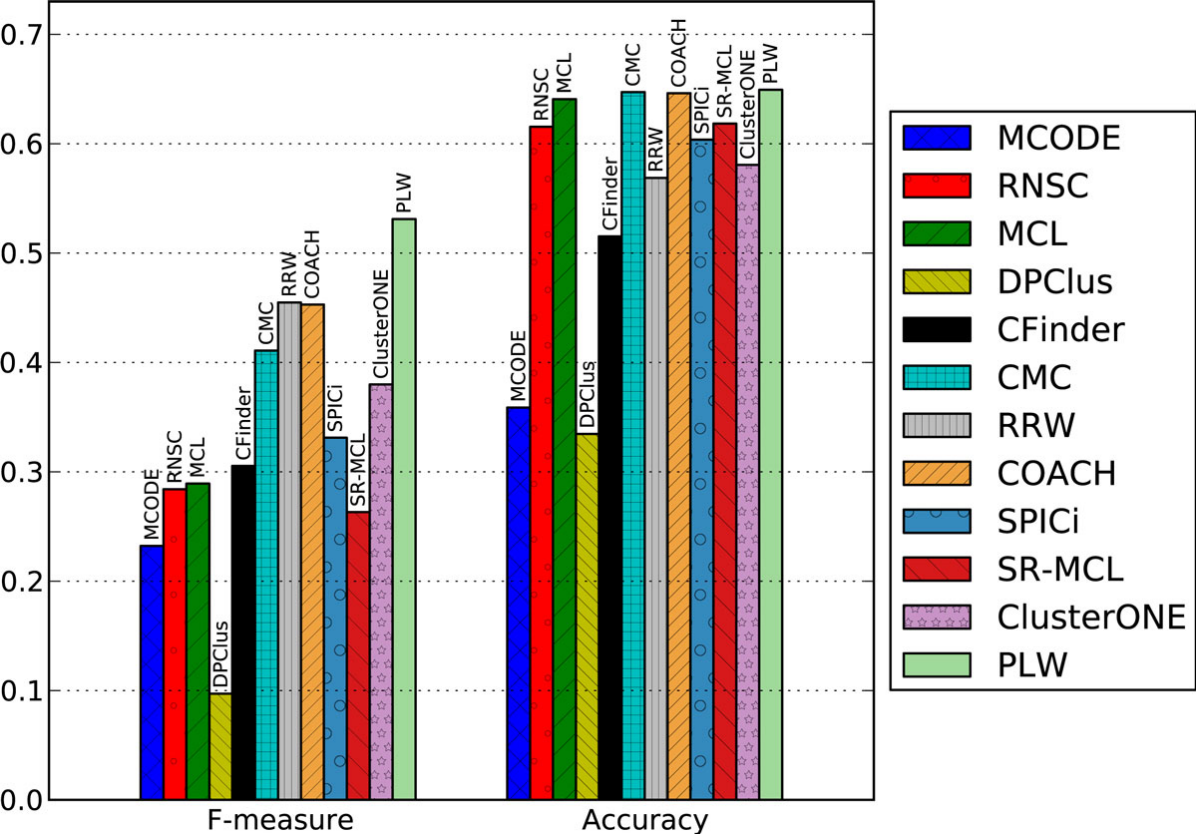
**Comparative performance of various methods on the DIP dataset using CYC2008 as benchmark**. The methods are ordered chronologically by the years in which they were published. Here, F-measure is the harmonic mean of *Recall *and *Precision*, whereas Accuracy is the geometric mean of *Sn *and *PPV*.

PLW achieved the highest F-measure compared to the other algorithms across all four combinations of the two PPI datasets and the two gold standards for protein complexes. In Figure 4, we present the F-measure and geometric accuracy of various algorithms on the DIP dataset evaluated using the CYC2008 benchmark. PLW attained the highest F-measure of 0.531, which is 16.7% (i.e. $\frac{0.531-0.455}{0.455}$) and 17.2% higher than the next highest of 0.455 for RRW and 0.453 for COACH, respectively. Meanwhile, PLW achieved a higher level of precision than other methods, indicating that more of our predicted protein complexes can be matched to benchmark complexes.

PLW's geometric accuracy is the highest as depicted in Figure 4 as a result of its high PPV and respectable sensitivity scores. The high PPV means that our method has a high proportion of correctly identified proteins in each predicted protein complex, which is consistent with the precision as analysed above.

Table 2 shows some statistics of complexes predicted by various algorithms, e.g., the number of predicted complexes (2*^nd ^*column), the average size of complexes (3*^rd ^*column) and the number of proteins covered (4*^th ^*column).

**Table 2:** Results of various algorithms on the DIP PPI network using CYC2008 as benchmark.

Algorithm	No. of Complexes	Average Complex Size	No. of Covered Proteins	*N_cb_*	*N_cp_*
MCODE	58	13.0	482	35	31
RNSC	541	3.87	667	119	107
MCL	600	6.84	801	126	119
DPClus	301	26.7	663	25	27
CFinder	245	10.2	1032	75	72
CMC	423	7.39	945	144	131
RRW	248	5.69	613	120	102
COACH	746	8.04	865	156	257
SPICi	412	5.13	700	118	102
SR-MCL	3879	13.6	1202	177	619
ClusterONE	342	4.84	596	103	115
PLW	576	6.03	782	149	264

Note that predicted clusters of two or less proteins are removed. For comparison, the average size of complexes in the CYC2008 benchmark is 6.68 proteins.

In addition, the comparison results on the other 3 combinations (i.e. COMBINED6 + CYC2008, DIP + NewMIPS and COMBINED6 + NewMIPS) are shown in Additional file 1.

### Benefits of seed selection strategy

In this experiment, we validate our hypothesis that selecting proteins in dense regions that have high degree centrality as seeds for expansion increases the precision of our algorithm. In addition, we apply our seed selection strategy to three other algorithms, namely, COACH [17], RRW [16] and ClusterONE [35]. By default, COACH and RRW use every protein as a seed for expansion, while ClusterONE keeps using the next unused protein seed with highest degree. For RRW, we show results using both a minimum cluster size of 5 (authors' default) and 3 (for a fairer comparison on par with other algorithms). This is justified since 32.1% (131 of 408) of gold standard complexes in the CYC2008 catalogue are of size 3 and 4.

**Figure 5:**
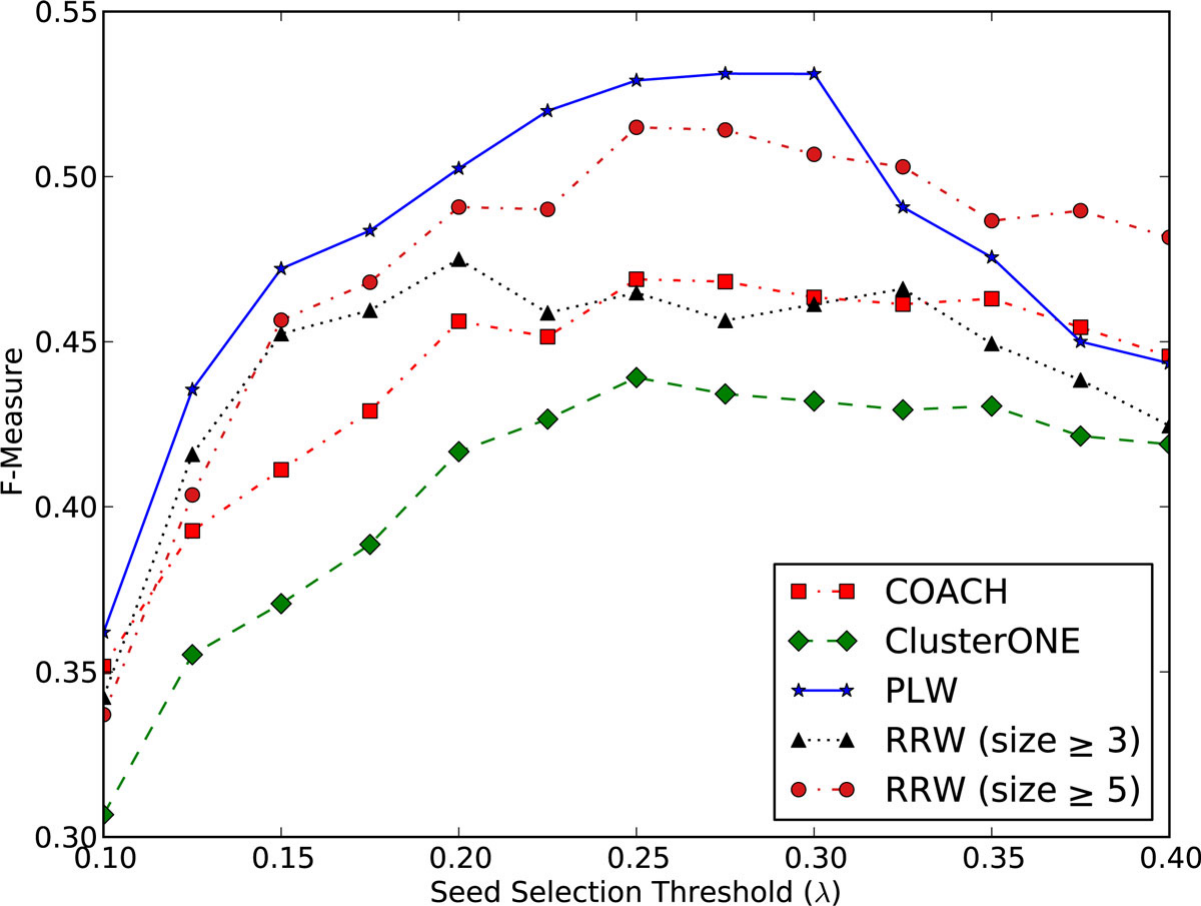
**F-measure against Seed Selection Threshold (λ) for PLW, RRW, COACH and ClusterONE**. λ is the fraction of the number of seeds over the total number of proteins present in the PPI graph. For each value of λ, we supplied the same set of seeds to all the algorithms. For RRW, we show results using a minimum cluster size of 5 (authors' default threshold) and 3 (for a fair comparison since most protein complex prediction algorithms predict complexes of size 3 and above).

For COACH, RRW and ClusterONE, their F-measure is **0**.**463**, **0**.**507 **and **0**.**432 **when λ is set as 0.3, as shown in Figure 5. They have even higher F-measure when λ is set as 0.25, e.g., **0**.**468 **for COACH, **0**.**515 **for RRW and **0**.**439 **for ClusterONE. Without the seed selection strategy, the F-measure for COACH, RRW and ClusterONE is **0**.**453**, **0**.**455 **and **0**.**380**, respectively. It is evident that our seed selection strategy enhanced the performance of existing algorithms for predicting protein complexes.

For the DIP dataset, PLW generates 118, 320, 576 and 787 clusters under λ = 0.1, 0.2, 0.3 and 0.4 respectively. With more seeds available as starting points for expansion into cores, the number of possible clusters increases thus explaining this trend.

**Figure 6:**
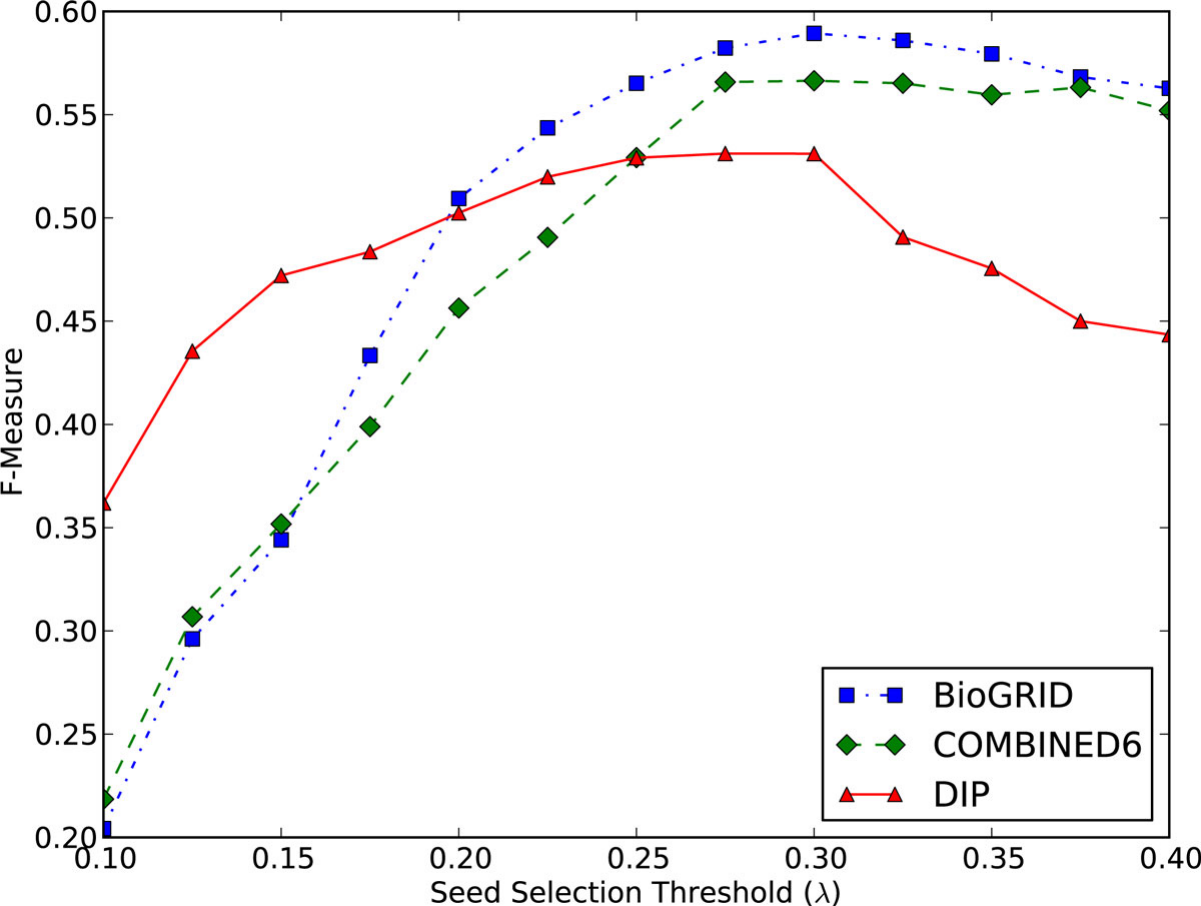
**F-measure against Seed Selection Threshold (λ) for PLW on DIP, COMBINED6 and BioGRID datasets**. F-measure is maximised at λ = 0.3 for all three PPI datasets.

We recommend the use of λ = 0.3 for PLW. This value yields high precision while allowing a reasonable rate of recall, as quantified by the peak in F-measure in Figure 5. This value also works well for other PPI datasets, as evidenced by the peak in F-measure at λ = 0.3 for all three datasets in Figure 6.

### Usefulness of common neighbour similarity

Common neighbour similarity is important for PLW's prediction of protein-complex cores, since it enables PLW to select protein pairs with high functional similarity.

**Figure 7:**
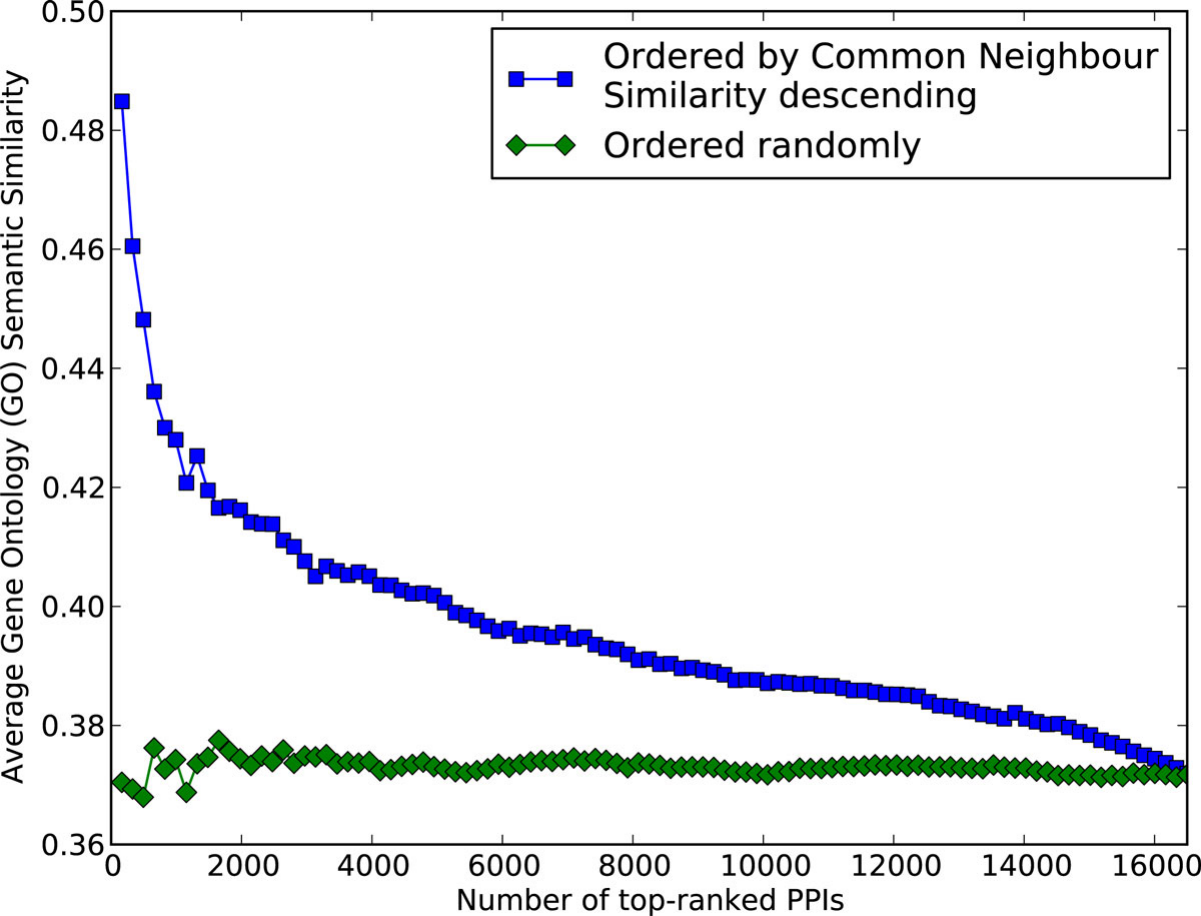
**Average Gene Ontology (GO) semantic similarity of PPIs ranked by their common neighbour similarity and those selected randomly, respectively**. We sorted pairs of interacting proteins by their common neighbour similarity and calculated the average GO semantic similarity for the top *x *protein interactions for *x *= 1, 2, ..., *|E_ppi_|*.

Our experiment in Figure 7 showed that picking protein pairs (i.e. protein interactions) with high common neighbour similarity yielded significantly higher functional similarity when compared to randomly picking the same number of protein pairs. This demonstrates the effectiveness of common neighbour similarity in estimating functional similarity. Functional similarity was quantified using Gene Ontology (GO) semantic similarity [46], with the terms in the Biological Process (BP) sub-ontology as it is the most informative (e.g., containing the most number of GO terms) [47].

**Figure 8:**
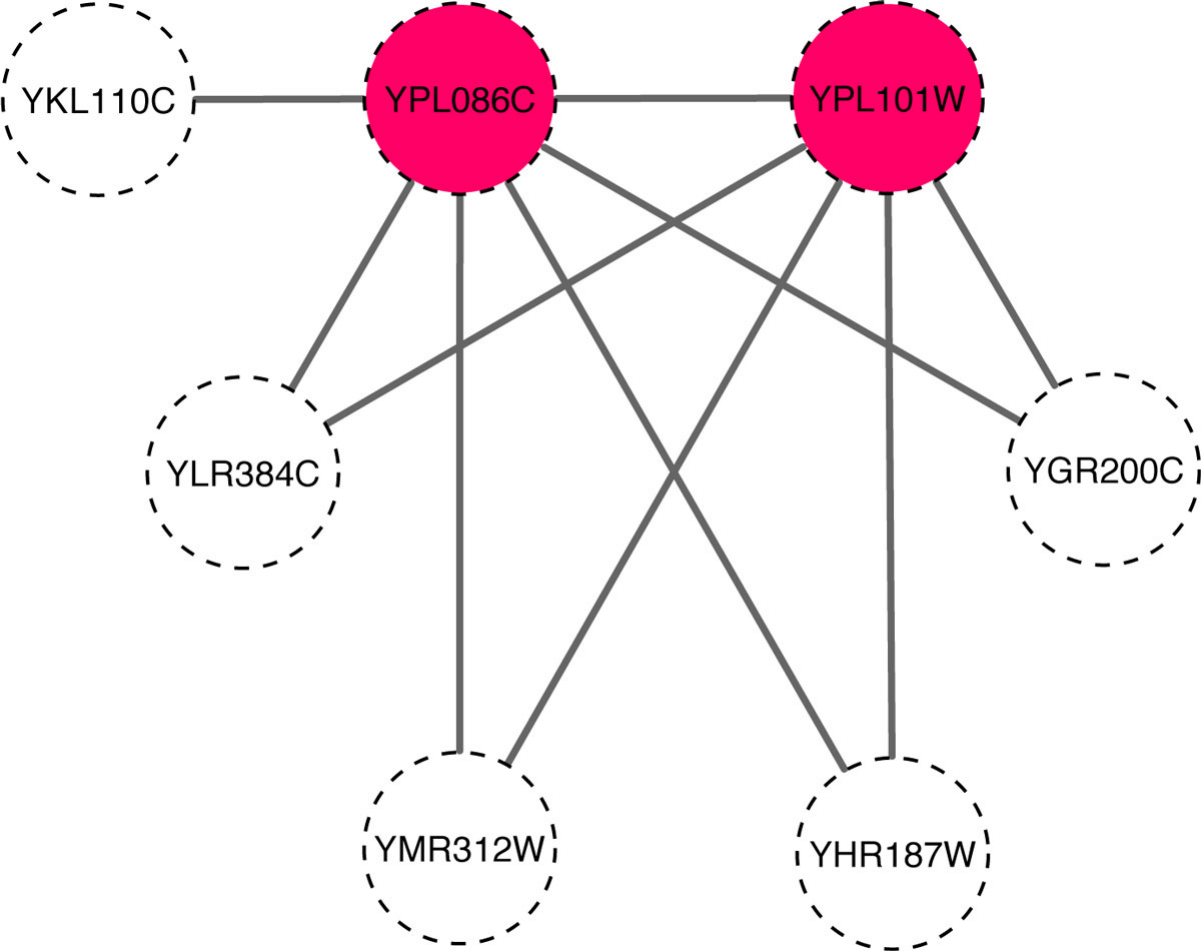
**Illustration of Common Neighbour Similarity in a PPI graph**. The two highlighted proteins, YPL08C and YPL101W, have a high number of common neighbours (4 proteins) and thus a high common neighbour similarity of $\frac{2+2}{\sqrt{\left(6+1\right)\left(5+1\right)}}=0.925$.

Figure 8 shows two interacting proteins, YPL086C and YPL101W, which have a high common neighbour similarity of 0.925. They have 6 and 5 neighbours and share 4 common neighbours, namely, YHR187C, YGR200C, YLR384C and YMR312C. YPL086C and YPL101C have a GO semantic similarity of 1 as they are members of the Elongator complex and share GO terms including "regulation of transcription from RNA polymerase II promoter" (GO:0006357) and "tRNA wobble uridine modification" (GO:0002098). Another example is the protein pair YLR170C and YPR029C. They have a high common neighbour similarity of 0.845 and are members of the AP-1 adaptor complex. They also share common GO terms, such as "Golgi to vacuole transport" (GO:0006896) and "vesicle-mediated transport" (GO:0016192). These two biological examples demonstrate that common neighbour similarity is useful for determining the functional similarity of two proteins.

### Co-localisation scores of predicted complexes

As the gold standard sets are incomplete [48], unmatched complexes could be undiscovered complexes. Colocalisation scores quantify the quality of these complexes by measuring the percentage of proteins in each complex that share a common localisation annotation [36,49]. This utilises the fact that a protein complex can be formed only when its constituents are found in the same cellular component [50]. PLW achieved high average co-localisation scores of 73% and 80% for the DIP and COMBINED6 datasets respectively, showing that it is able to detect biologically relevant protein complexes.

### Biological case studies

In this section, we conduct a qualitative analysis of the protein complexes predicted by our PLW algorithm. PLW was able to detect 16 benchmark complexes in the CYC2008 gold standard with better accuracy than existing methods.

**Figure 9:**
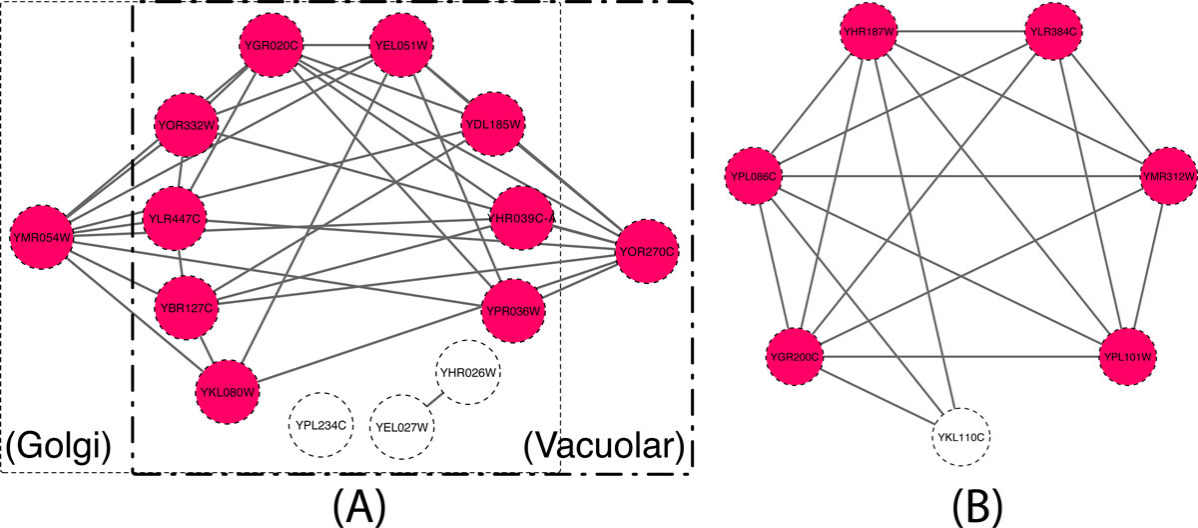
**Examples of benchmark protein complexes predicted more accurately by PLW**. The coloured proteins are those recovered by PLW while the white proteins are missed. (A) shows two overlapping complexes, with the left thin-dotted box showing the H+-transporting ATPase (Golgi) complex and the right thick-dotted box showing the H+-transporting ATPase (Vacuolar) complex. (B) shows the DNA replication factor C complex (Ctf18p/Ctf8p/dcc1p) complex. PLW did not include any proteins outside of the benchmarks.

In Figure 9, we show two examples that were detected with higher accuracy by PLW. Figure 9(A) shows two overlapping complexes, H+-transporting ATPase (Golgi) and H+-transporting ATPase (Vacuolar). The complex predicted by PLW consists of **11 **proteins, covering **11 **proteins in the benchmark complex. The next best match was by ClusterONE with **9 **proteins, which did not recover the proteins YDL185W and YLR447C. (Figure 9(B) shows our predicted complex that matches "DNA replication factor C complex (Ctf18p/Ctf8p/dcc1p)" in CYC2008 (with neighbourhood affinity score **0**.**69**). The next best match was generated by RRW, whose predicted complex has 5 proteins and recovers 4 proteins in the real complexes (with neighbourhood affinity score **0**.**56**). Additionally, the two protein complexes detected only by PLW were the box C/D snoRNP complex (4 proteins) and ISW1b complex (3 proteins), which were matched with neighbourhood affinity scores of 0.25 and 0.33, respectively.

PLW is able to recover the complexes with high accuracy, as shown in Figure 9. Therefore, we believe that PLW will be useful to biologists in predicting high quality protein complexes for further investigation.

## Conclusions

As experimental protein complex detection remains a challenging problem, it is important to develop accurate computational approaches for predicting protein complexes from PPI data. The continued explosion in the volume of available PPI data demands more efficient and more precise algorithms. We used our PLW algorithm to demonstrate three techniques, which can also be applied to improve the performance of other protein complex prediction algorithms and even general graph clustering algorithms. These techniques are:

1. **A precise and efficient Probabilistic Local Walks (PLW) algorithm for mining protein complex cores**. PLW attained the best F-measure (recall and precision), with an improvement of 16.7% over the next best method amongst the 11 methods evaluated. It carries out probabilistic local walks to mine cores efficiently in $O\left(\left|V|\text{log}|V|+|E\right|\right)$ time. This efficiency renders it competitive on larger PPI networks (e.g., human) on which other algorithms are unable to compete.

2. **Seed selection strategy**. We developed a scoring strategy that finds important seeds to expand without excluding important proteins or including too many harmful seeds. This strategy yielded increased precision for PLW, COACH, RRW and ClusterONE.

3. **Common neighbour similarity**. We formulated a measure to estimate the functional similarity of two proteins using their common neighbours. We found that common neighbour similarity is highly correlated with functional similarity, rendering it useful in detecting complexes with functional homogeneity. In addition, common neighbour similarity can be applied in situations where functional information is not readily available.

For future work, we are exploring how to automatically determine a suitable value for the threshold λ in the seed selection strategy to increase its applicability to the large range of agglomerative clustering algorithms. We are also studying the mathematical properties of PLW's novel walking method.

The techniques we conceived will be useful for researchers in graph clustering. In particular, PLW could be applied to cluster other biological networks, such as metabolic networks and gene regulatory networks. In addition, PLW could be parallelised to tackle massive networks. We will explore such applications as our future work.

## Competing interests

The authors declare that they have no competing interests.

## Authors' contributions

DLKW, XLL and MW conceptualised and designed the method, and drafted the manuscript together. DLKW was responsible for the implementation and carried out the experiments. JZ and SKN participated in discussion as well as revision of the draft. All authors read and approved the manuscript.

## Supplementary Material

Additional file 1 **Performance of algorithms on various datasets.pdf**. This file contains four figures comparing the algorithms' performance on the following datasets and gold standards: 1. DIPS PPI dataset against CYC2008 gold standard, 2. DIPS PPI dataset against NEWMIPS gold standard, 3. COMBINED6 PPI dataset against CYC2008 gold standard and 4. COMBINED6 PPI dataset against NEWMIPS gold standard. Click here for file

## Declarations

Publication of this article was funded by the Institute for Infocomm Research, Agency for Science, Technology and Research (A*STAR), Singapore.

This article has been published as part of *BMC Genomics *Volume 14 Supplement 5, 2013: Twelfth International Conference on Bioinformatics (InCoB2013): Computational biology. The full contents of the supplement are available online at http://www.biomedcentral.com/bmcgenomics/supplements/14/S5.
