# Supplementary material for: PLW: Probabilistic Local Walks for detecting protein complexes from protein interaction networks
Source: BMC Genomics. 2013 Oct 16;14(Suppl 5):S15. doi: 10.1186/1471-2164-14-S5-S15 (PMC3852146; doi:10.1186/1471-2164-14-S5-S15)

Supplementary File 1 - Performance of algorithms on DIPS data against CYC2008 gold standard

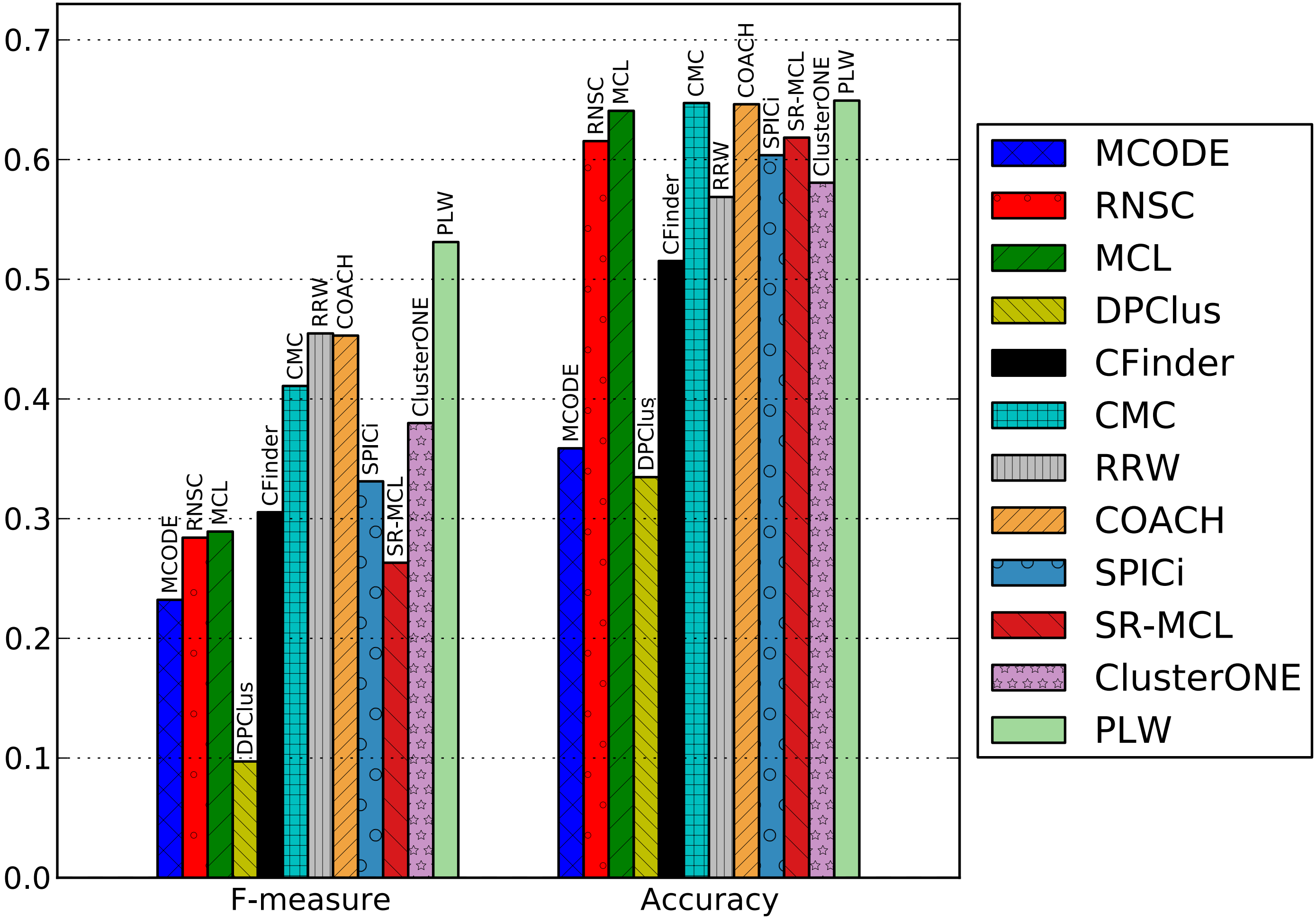

Supplementary File 2 - Performance of algorithms on DIPS data against NEWMIPS gold standard

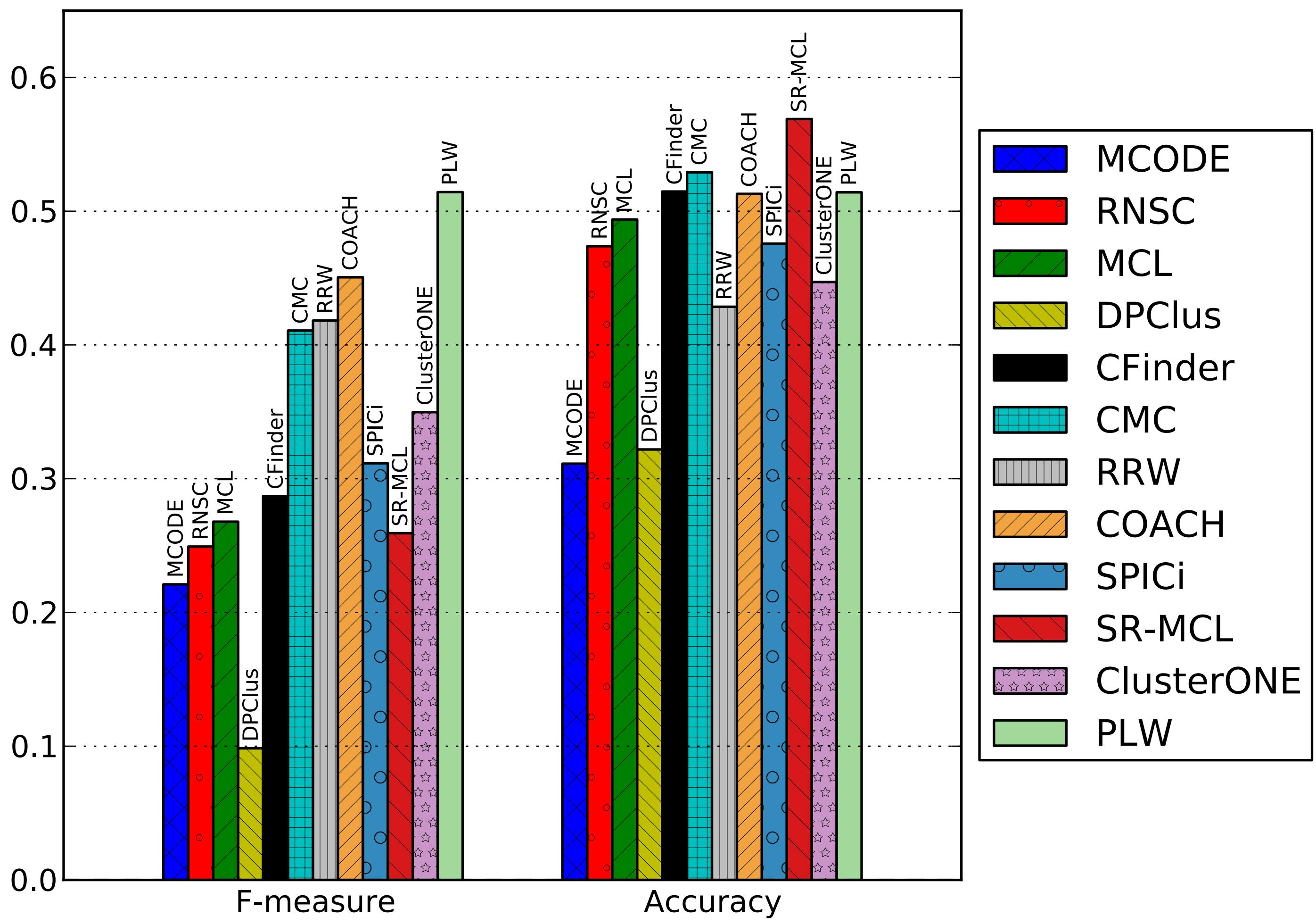

Supplementary File 3 - Performance of algorithms on COMBINED6 data against CYC2008 gold standard

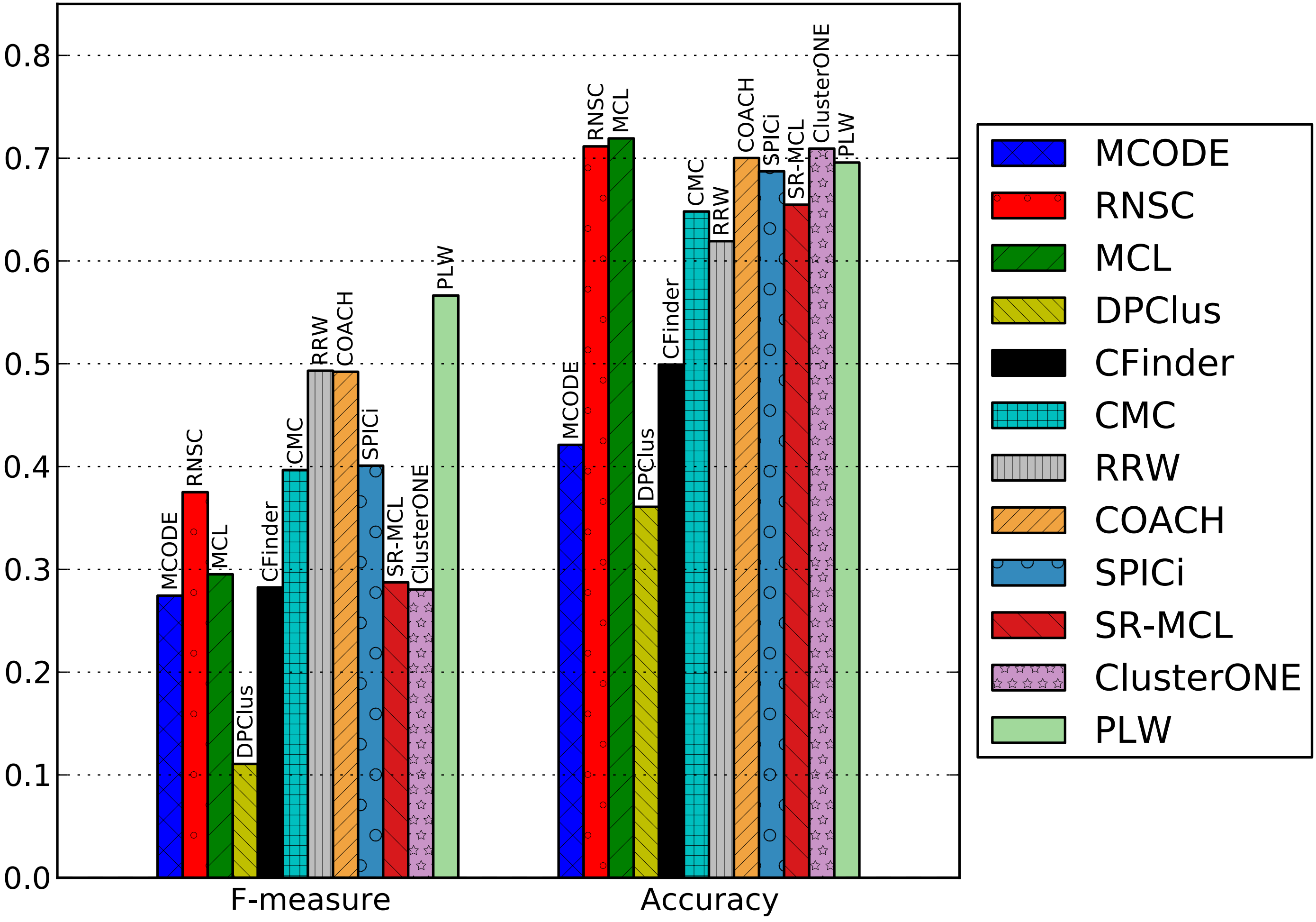

Supplementary File 4 - Performance of algorithms on COMBINED6 data against NEWMIPS gold standard

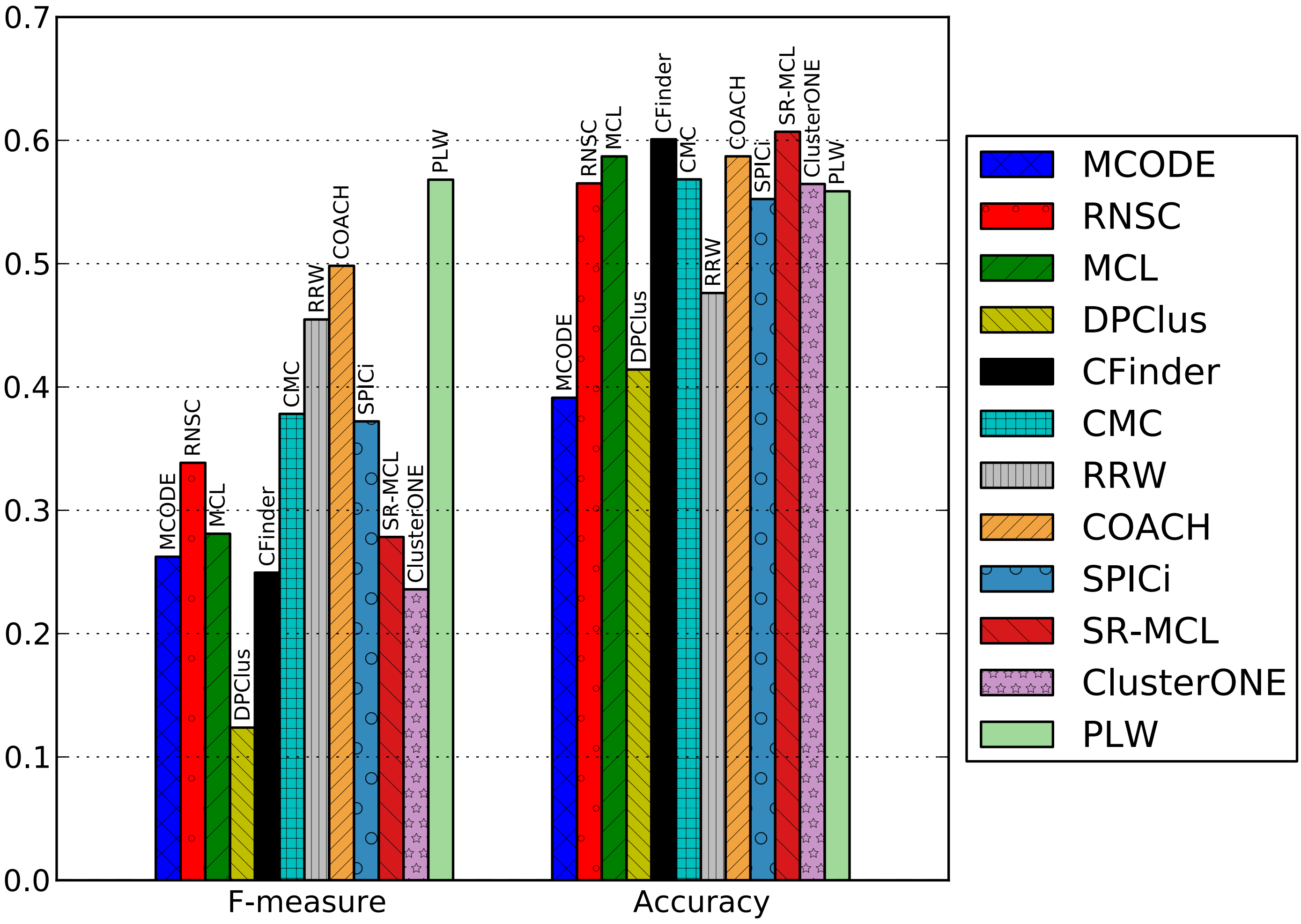

Supplement: Additional file 1 — Performance of algorithms on various datasets.pdf. This file contains four figures comparing the algorithms' performance on the following datasets and gold standards: 1. DIPS PPI dataset against CYC2008 gold standard, 2. DIPS PPI dataset against NEWMIPS gold standard, 3. COMBINED6 PPI dataset against CYC2008 gold standard and 4. COMBINED6 PPI dataset against NEWMIPS gold standard. [file 1471-2164-14-S5-S15-S1.pdf]
